# Correlations of polyploidy and apomixis with elevation and associated environmental gradients in an alpine plant

**DOI:** 10.1093/aobpla/plw064

**Published:** 2016-10-26

**Authors:** Christoph C. F. Schinkel, Bernhard Kirchheimer, Agnes S. Dellinger, Simone Klatt, Manuela Winkler, Stefan Dullinger, Elvira Hörandl

**Affiliations:** 1 Department of Systematics, Biodiversity and Evolution of Plants (with herbarium), Georg-August-University of Göttingen, Untere Karspüle 2, 37073 Göttingen, Germany; 2 Department of Botany and Biodiversity Research, University of Vienna, Rennweg 14, 1030 Vienna, Austria; 3 GLORIA co-ordination, University of Natural Resources and Life Sciences Vienna, Centre for Global Change and Sustainability, Vienna, Austria

**Keywords:** Apomixis, environmental gradients, fitness, flow cytometry, geographical parthenogenesis, polyploidy, *Ranunculus kuepferi*

## Abstract

Apomictic plants expand their geographical distributions more to higher elevations compared to their sexual progenitors. It was so far unclear whether this tendency is related to mode of reproduction itself or represents a side effect of polyploidy. Apomixis is advantageous for range expansions as no mating partners and pollinators are needed (Baker’s rule). Polyploidy is thought to infer fitness advantages and a higher vigour that would enable plants to adjust better to more extreme climatic conditions. However, little is known about actual performance of plants at higher elevations. We analyzed 81 populations of *Ranunculus kuepferi* from the whole distribution area in the European Alps to quantify apomictic versus sexual seed formation via flow cytometric seed screening. Seed set and vegetative growth were measured as fitness parameters. All parameters were correlated to geographical distribution, elevation, temperature and precipitation. Flow cytometric seed screening revealed predominantly obligate sexuality (88.9 %) and facultative apomixis in diploid populations, while tetraploid populations are predominantly facultative (65.4 %) to obligate apomictic. Apomictic seed formation correlated significantly to higher elevations, which explains also the observed niche shift to lower temperatures. However, within the tetraploid range, there is no apparent correlation of degree of facultative apomixis to geographical distance. Apomixis appeared in diploids three times independently in separated, otherwise sexual populations in the southwestern refugial areas of the Alps. Diploid apomixis was not successful in range expansions, and obligate sexual polyploids were not observed. Polyploidy may relate to cold tolerance as an adaptation to conditions at high elevations, where diploid sexuals have no fitness advantage. Instead, facultative apomixis may have aided colonization of higher elevations and range expansions in the Alps without mate and pollinator limitation, but did not necessarily involve long-distance dispersal. A direct influence of low temperatures on unreduced gamete formation cannot be ruled out.

## Abstract (precis)

We are presenting a study on geographical parthenogenesis, an enigmatic and much disputed phenomenon. Based on a large sampling of natural populations of Ranunculus kuepferi, it is the first quantitative, population-based assessment of mode of reproduction throughout the Alps to test for correlations to elevation and to geographical distance patterns. Surprisingly, we found a high variance in the modes of reproduction among cytotypes and can give the first evidence of apomixis in diploid natural populations. Further, a significant correlation of ploidy to elevation was found, as well as the correlations of mode of reproduction to environmental gradients.

## Introduction

The term geographical parthenogenesis (GP) denotes a long known phenomenon that closely related sexual and apomictic taxa exhibit largely divergent distribution patterns (Vandel, 1928). A number of recent studies have dealt with aspects of GP both in plants and animals. Distribution areas of asexual organisms generally seem to be larger than those of their sexual congeners, mostly expressing a tendency to marginal habitats such as higher latitudes and elevations as well as to disturbed areas (Bell, 1982; Bierzychudek, 1985; Van Dijk, 2003; Kearney, 2005; Hörandl, 2006, 2009). However, the causal reasons for these shifts in distribution patterns are only poorly understood and have given rise to competing hypotheses.

Apomicts inherently possess the potential of founding new populations by only a single propagule or individual. Unlike their sexual relatives, apomictic plants do not necessarily require any pollinators or mating partners because they generate their seeds either independently of pollen (autonomous endosperm) or are able to use self-pollen for fertilization of the endosperm (pseudogamy); Hörandl (2010). Similar to the advantages of selfing, such uniparental reproduction is described in Baker’s law as beneficial for colonization, and is expected to be most efficient after long-distance dispersal (Baker, 1965, 1967; Pannell *et al.*, 2015).

Although Baker’s law explains well horizontal range expansions of apomictic plants (Baker, 1955, 1967; Mogie and Ford, 1988; Cosendai *et al.*, 2013), it does not provide a theoretical background for the strong tendency of apomicts to colonize higher elevations and latitudes (Bierzychudek, 1985; Asker and Jerling, 1992). Alpine zones in high mountain systems represent extreme habitats for plant life, with lower temperatures including freezing, shorter vegetation periods and stronger wind exposure with increasing elevation (Nagy and Grabherr, 2009). Under this aspect, different lines of hypotheses can be developed to specifically explain a preference of apomicts for higher elevations: first, apomictic plants are mostly polyploids (Carman, 1997), and polyploidy by itself confers genomic features which could provide more physiological and ecological flexibility to adapt to harsh conditions. Polyploidy is characterized by higher allelic diversity, heterozygosity and often by heterosis effects (Birchler *et al.*, 2010). Genome doubling can be accompanied by gene silencing, diversification in gene expression, differential expression of gene copies and epigenetic changes (Osborn *et al.*, 2003; Adams and Wendel, 2005; Comai, 2005; Hegarty and Hiscock, 2008). Epigenetic change and alterations in gene expression are important for the control of phenotypic plasticity and rapid adaptation (Nicotra *et al.*, 2010). Accordingly, benefits of polyploidy could indirectly promote adaptation of apomicts to higher elevations (Bierzychudek, 1985). However, little is known about reproductive fitness and vegetative performance under extreme alpine conditions (Wagner and Reichegger, 1997; Wagner and Mitterhofer, 1998; Kudo and Hirao, 2005; Ladinig *et al.*, 2013).

Second, a preference for extreme habitats could be explained by population genetic structure in apomictic taxa. The frozen niche variation model (FNV) described by Vrijenhoek (1984, 1994; Vrijenhoek and Parker, 2009) postulates an advantage of apomicts based on their efficiency in niche exploitation. The model assumes that hybrid origin, autopolyploidization and facultative sexuality among the descendants constitute multiple discrete arrays of clones featuring different and diverse genotypes. Natural selection causes partitioning among the clonal lineages and accordingly, some apomictic genotypes may specialize on ecological niches outside the ecological optimum of the sexual parent(s). Clonal arrays do not only encompass most of the niche space of the sexual progenitor(s), but can also exploit the extreme range of the niche space more efficiently (Vrijenhoek and Parker, 2009).

Beside these ‘classical’ explanations, the direct influence of climate is also brought into focus of the discussion. Cold stress is known to trigger formation of unreduced pollen (Ramsey and Schemske, 1998; Bomblies *et al.*, 2015), which is due to disturbance of microtubule formation at meiosis and defects in post-meiotic cytokinesis (De Storme *et al.*, 2012). Unreduced pollen formation, however, is the major pathway leading to sexual polyploidization (De Storme *et al.*, 2013). Female development, where the same principles may apply for the formation of unreduced embryo sacs, is even less understood because of methodological difficulties to study female meiosis. Furthermore, there is a lack of experimental work and quantitative data on these processes in natural populations of non-model species under alpine conditions.

The alpine species *Ranunculus kuepferi* is a suitable model system for studying the correlations of mode of reproduction, polyploidy and elevation. The species has diploid and tetraploid cytotypes, with diploids occurring in the southwestern parts of the Alps, while tetraploids colonize the Northern, Central and Eastern Alps, Corsica and the Northern Apennines (Burnier *et al.*, 2009; Cosendai and Hörandl, 2010). Post-glacial colonization of the Alps happened probably out of the southwestern glacial refugia (Burnier *et al.*, 2009). Previous analyses of spot samples on a few individuals suggested sexual seed formation in diploids, and facultative apomixis in tetraploids (Burnier *et al.*, 2009; Cosendai and Hörandl, 2010). However, these studies did not quantify pathways of seed formation, and hence could not test for statistical correlations to geographical distances, elevation and related environmental parameters. Population genetic studies suggested autopolyploid origin (Cosendai *et al.*, 2011), a high genotypic diversity and lack of geographical structure among tetraploids (Cosendai *et al.*, 2013). Self-fertility of tetraploids supported the assumption of Baker’s law that rapid colonization could have played a role in distributions (Cosendai *et al.*, 2013). Recently, Kirchheimer *et al.* (2016) found a niche shift between diploid and tetraploid cytotypes, mostly towards lower temperatures, but it remained unclear whether this shift actually correlates to mode of reproduction or to other physiological features connected to polyploidy. Shifts to lower temperatures in high elevations and northern latitudes are often connected to special morphological adaptations, like small growth form (alpine dwarfism), which is mostly due to slower cell cycle and cell differentiation processes (Körner, 2003). Shifts to higher elevations, however, are also connected to additional physiological stress factors, like higher UV radiation, and lower CO_2_ atmospheric pressure, resulting in lower carbon availability (Körner, 2003).

So far, it was unknown whether ploidy levels and mode of reproduction are strictly correlated, or whether sexual tetraploid or apomictic diploid plants do occur in natural populations. Kirchheimer *et al.* (2016) hypothesized that the observed niche shift may not be the decisive factor, but rather consequence of a rapid colonization process, which was enhanced by the ability of rapidly founding populations via apomixis. In the case of frequent founder events after long distance dispersal, apomictic seed production should be most frequent in the marginal populations of the distribution range. If the colonization process was mainly driven by the niche shift according the Frozen Niche Variation model, frequencies of apomixis should be positively correlated to the coldest locations, i.e. either in elevation or in latitude. However, no study has so far quantitatively compared the proportions of facultative sexual reproduction within and among populations at different elevations and in different parts of the distribution range. Moreover, the full range of pathways of seed formation possible in apomictic plants (Matzk *et al.*, 2000; Dobeš *et al.*, 2013), had never been assessed before in *R. kuepferi*. For instance, it was so far unknown to which extent tetraploid obligate sexuals or diploid apomicts would contribute to the distribution patterns. Here, we present a comprehensive dataset on reproductive pathways and fitness parameters of *R. kuepferi* from 81 populations out of the European Alps to test the following hypotheses: (1) Are mode of reproduction and ploidy level strictly correlated? (2) Is diploid apomixis or sexual polyploidy successful in range expansions? (3) Is there a correlation of apomictic mode of reproduction to higher elevation, or do we find a correlation to geographical distance? (4) Are there quantitative differences in seed set and in morphological fitness between cytotypes, and do they correlate to elevation? (5) Are there correlations of mode of reproduction to key climatic factors at higher elevations, i.e. temperature and precipitation? A more comprehensive study on the effects of climate factors and niche dynamics on cytotypes has been presented elsewhere (Kirchheimer *et al.*, 2016).

## Methods

### Plant material

Plants of Kuepfer’s buttercup (*Ranunculus kuepferi*) have been collected throughout the Alps from 81 populations during two consecutive summer periods in 2013 and 2014 **[see Supporting Information—Table S1]**. We accessed all published localities (Burnier *et al.*, 2009; Cosendai and Hörandl, 2010) as well as records from herbaria and from the floristic literature. At the sites, we randomly selected a 100 m × 100 m plot to define a population. Apart from three exceptions where less than five plants were found, we sampled 12 individuals per population (1074 in total) in the post-anthesis to the early fruiting stage. Microscopic investigations on ovule development (Burnier *et al.*, 2009; C. Schinkel unpubl. data, following methods of Hojsgaard *et al.*, 2014) confirmed that ovule development happens in *R. kuepferi* during the very early bud stage. All buds collected in the wild already showed fully mature female gametophytes and represent the 7-celled, 8 nucleate Polygonum-type embryo sac, as typical for *Ranunculus* (Nogler, 1984; Hojsgaard *et al.*, 2014). This fits to general observations that alpine plants produce floral primordia in the year before, and finish ovule development in buds below ground before sprouting (Körner, 2003; Nagy and Grabherr, 2009). Hence, we can assume that sexual vs. apomictic developmental pathways in *R. kuepferi* were already completed under natural conditions before collection of plants, and only ripening of seeds happened under garden conditions. Plants were taken from four 2 m × 2 m randomly chosen subplots (Kirchheimer *et al.*, 2016). All plants were dug out, transported to the Botanical Garden of the University of Göttingen, and cultivated in pots. Single fruiting heads were bagged with perforated plastic pouches to harvest all mature achenes of a collective fruit. Achenes were kept for at least 10 days at room temperature, before bundled in paper bags and stored on silica gel at 8 °C for later analyses.

### Flow cytometric seed screen (FCSS) and ploidy determination

Like many other facultative apomicts, a single plant can produce both sexual and apomictic seeds within the same flower (Aliyu *et al.*, 2010; Dobeš *et al.*, 2013). To quantify the main mode of reproduction, we determined ploidies of both endosperm and embryo per single seed for each individual. Since many tetraploid plants had a poor seed set, as reported previously (Huber, 1988; Cosendai and Hörandl, 2010), we had to restrict the sampling to 551 individuals, which formed each a minimum of five well-developed seeds per flower. Five seeds per plant from at least three plants per population were analyzed with a slightly modified FCSS method according to Matzk *et al.* (2000). Seeds were placed in 2 ml Eppendorf tubes together with two 0.23 cm steel beads (QIAGEN, Hilden, Germany) and ground in a TissueLyser II mill (QIAGEN, Hilden, Germany) with a stroke rate of 30 Hz for 7 s. Further preparation was realized using a two-step procedure described by Doležel *et al.* (2007) performing (1) a nuclei isolation step with Otto I buffer: 0.1 M citric acid monohydrate, 0.5 % v/v Tween 20 (Sigma-Aldrich Munich, Germany), ddH_2_O and (2) a separate staining step with Otto II buffer: 0.4 M Na_2_HPO4, ddH_2_O and charged with 3 ng/ml 4′,6-diamidinophenyl-indole (Sigma-Aldrich, Munich, Germany). Macerated seeds were incubated for 5 minutes with 200 µl ice-cold Otto I buffer. Suspensions were filtered through 40 µm mesh tubes (Partec, Münster, Germany). 800 µl Otto II buffer were then added and incubated for another 15 minutes before analysis. Ploidy levels of all mother plants were determined on fresh leaves from the cultivated plants using the same methods as described above, except for a slightly prolonged grinding time in the TissueLyser (15 s).

All analyses were performed on a CyFlow Space flow cytometer (Partec, Münster, Germany). Histograms were taken and analyzed with the supplied FloMAX Software version 2.2.0 (Quantum Analysis GmbH, Münster, Germany). Leaf material of *Zea mays* (CE-777 strain, provided by Doležel J.) and a diploid tested plant of *R. kuepferi* were used as external reference standard to adjust the gain level of the UV LED lamp. All subsequent analyses were conducted with the same parameters.

Peak ranges for embryo (em) and endosperm (es) were set manually in FloMAX and values of DNA content were calculated as Gaussian means. Ratios of es: em ploidies were calculated to determine whether a seed has been produced sexually (3:2 ratio) or via apomixis (3:1, 2.5:1, 2:1 ratio). Interpretation of all plausible pathways for development and fertilization of seeds of *R. kuepferi* (Table 1) have been adopted from the studies by Matzk *et al.* (2000), Talent and Dickinson (2007), Cosendai and Hörandl (2010) and Dobeš *et al.* (2013), and provided the basis for our classification; terminology for designation of ploidy levels follows Greilhuber *et al.* (2005). A threshold of 1.65 es:em ratio was set to discriminate between sexual (lower values) and asexual (higher values) cases. Those with ratio values between 1.85 and 2.15 were interpreted as autonomous endosperm development since the second peak was always distinct and as high as the endosperm peak in other pathways. Hence, we interpreted it as endosperm peak, and we excluded the possibility that it could represent just a G2 peak of the growing embryo (G2 peaks are usually much smaller than the respective G1 peak, as only few cells are in the respective stage of the cell cycle). Representative flow cytometric histograms are shown in **Supporting Information–Figure S1**.

**Table 1:** Observed pathways of seed formation in *Ranunculus kuepferi*.

		N			Ploidy		Genome contribution of sperm nuclei to endosperm
		seed	polar nuclei	sperm nuclei	Embryo	Endosperm	
Diploid							
Sexual	A	663	2	1	1Cx(m) + 1Cx(p)	2Cx(m) + 1Cx(p)	1 reduced
BIII	AB	4	2	1 or 2	2Cx(m) + 1Cx(p)/2Cx(m) + 2Cx(p)	4Cx(m) + 1Cx(p)/4Cx(m) + 2Cx(p)	1 reduced/1 unreduced
	A2	30	2	1	2Cx(m)	4Cx(m) + 1Cx(p)	1 reduced
Asexual	A3	2	2	1 or 2	2Cx(m)	4Cx(m) + 2Cx(p)	2 reduced or 1 unreduced
	A4	2	2	2	2Cx(m)	4Cx(m) + 3Cx(p)	2 reduced (∼ 1.5Cx)*

Triploid							
Sexual	B	6	2	1	1Cx(m) + 2Cx(p)/2Cx(m) + 1Cx(p)	2Cx(m) + 2Cx(p)/4Cx(m) + 1Cx(p)	1 reduced (diploid/haploid sperm nuclei)
BIII***	BB	6	2	2	3Cx(m) + 1Cx(p)	6Cx(m) + 6Cx(p)/6Cx(m) + 4Cx(p)/6Cx(m) + 2Cx(p)	endosperm polyploidization, 2 reduced (diploid/haploid sperm nucleus)
	B2	101	2	1 or 2	3Cx(m)	6Cx(m) + 2Cx(p)/6Cx(m) + 1Cx(p)	1 reduced (diploid/haploid sperm nucleus)
	B3	12	2	1 or 2	3Cx(m)	6Cx(m) + 5Cx(p)/6Cx(m) + 4Cx(p)	2 reduced (∼ 2.5Cx)*/2 reduced
Asexual	B4	2	2	1 or 2	3Cx(m)	6Cx(m) + 3Cx(p)	1 unreduced or 2 reduced (∼ 1.5Cx)*
	B5	2	2	2	3Cx(m)	6Cx(m) + 6Cx(p)	2 unreduced
	D1	2	2	1	3Cx(m)	12Cx(m) + 3Cx(p)	endosperm polyploidization + 1 unreduced

Tetraploid							
Sexual	C	118	2	1	2Cx(m) + 2Cx(p)	4Cx(m) + 2Cx(p)	1 reduced
BIII	CB	33	2	1 or 2	4Cx(m) + 2Cx(p)/4Cx(m) + 4Cx(p)	8Cx(m) + 2Cx(p)/8Cx(m) + 4Cx(p)	1 unreduced/2 reduced
	C2	1258	2	1	4Cx(m)	8Cx(m) + 2Cx(p)	1 reduced
	C3	400	2	1 or 2	4Cx(m)	8Cx(m) + 4Cx(p)	2 reduced or 1 unreduced
	C4	58	2	2	4Cx(m)	8Cx(m) + 6Cx(p)	2 reduced (∼3Cx)*, **
Asexual	C5	24	2	2	4Cx(m)	8Cx(m) + 8Cx(p)	2 unreduced or endosperm poyploidization, **
	C6	16	2	0	4Cx(m)	8Cx(m)	autonomous endosperm
	D2	25	2	1	4Cx(m)	8Cx(m) + 1Cx(p)	1 reduced diploid
	D3	35	2	1 or 2	4Cx(m)	8Cx(m) + 3Cx(p)	1 reduced (∼3Cx)*
	D4	4	2	1	4Cx(m)	16Cx(m) + 4Cx(p)	endosperm polyploidization + 1 unreduced

Cx, ploidy after DNA content (Greilhuber *et al*., 2005); m, maternal genome contribution; p, paternal genome contribution; * after unbalanced pollen meiosis; ** also trinucleate endosperm possible (C4: 12Cm + 2Cp, C5: 12Cm + 4Cp; see also Talent and Dickinson 2007); *** only possible variants of observed cases presented.

We categorized every individual and population as obligate sexual (only sexual seeds), obligate apomictic (only apomictic seeds) or mixed (sexual as well as apomictic seeds = facultative apomixis) by pooling the results of the analyzed seeds. Since we had just a sampling of *n* = 5 seeds per individual, we did not calculate individual-level percentages, but just recorded the category for each plant. Instead, we pooled all seeds per population for calculating percentages and correlations of mode of reproduction with other variables.

### Seed set and morphological fitness parameters

Seed set was determined as percentage of all mature achenes per individual and measured as the total number of well-developed over the total number of achenes per flower per plant (Cosendai and Hörandl, 2010). Discrimination between filled and empty achenes was done manually using a forceps. Filled ones have a well-developed endosperm and withstand applied pressure, while undeveloped ones collapse easily.

As a measurement for individual vegetative fitness, we quantified number and length of leaves (longest leaf per rosette) and shoots as well as the number of buds, flowers and fruits of each plant before collection directly in the field (i.e. under natural conditions). Measurements served as a proxy for comparisons between cytotypes and for estimating the presence of heterosis in polyploids.

### Statistical analyses

All analyses were conducted in R version 3.1.2 (R Core Team, 2014) using the external packages R Commander (Rcmdr) version 2.1-5 (Fox, 2005) and lattice version 0.20-29 (Sarkar, 2008).

Percentages were arcsine transformed, all other variables were converted to the natural logarithm before analysis to improve normal distribution of the data. Seed set was determined per flower and subsequently averaged per plant. To identify effects of ploidy and selected ecological parameters (WorldClim) on distribution differences and reproductive mode variances between cytotypes, we conducted one-way ANOVA on population scale, with ploidy and the respective analysis dependent predictor as fixed effect. Seed set differences between cytotypes were equally analyzed with ANOVA, and ploidy as well as the respective ecological predictors as fixed effects. Further, correlations among ecological predictors and reproduction mode, seed set, ploidy were tested on population scale with Spearman’s rank-order correlation.

### Environmental factors

We tested correlations of elevation and several climatic variables at the sites of plant origin, i.e. our sampling sites, on cytotypes and modes of reproduction. Data for elevation were taken from the collection sites with the barometric altimeter of an eTrex 30 GPS device (Garmin Deutschland GmbH, Garching, Germany) (see Appendix A). Climatic variables were downloaded from WorldClim (www.worldclim.org) with a spatial resolution of approximately 1 km^2^ (Hijmans *et al.*, 2005). To fit climate data to our sample-plot size, WorldClim variables have been statistically downscaled to a resolution of 100 m × 100 m (for detailed description see Kirchheimer *et al.*, 2016). From the provided 19 bioclimatic variables within the dataset, we chose BIO1 as annual mean temperature, BIO7 as temperature annual range (maximum–minimum temperature), BIO10 as mean temperature of warmest quarter and BIO12 as annual precipitation—which represent the most important climatic drivers of plant growth—for our analysis.

## Results

**Figure 1:**
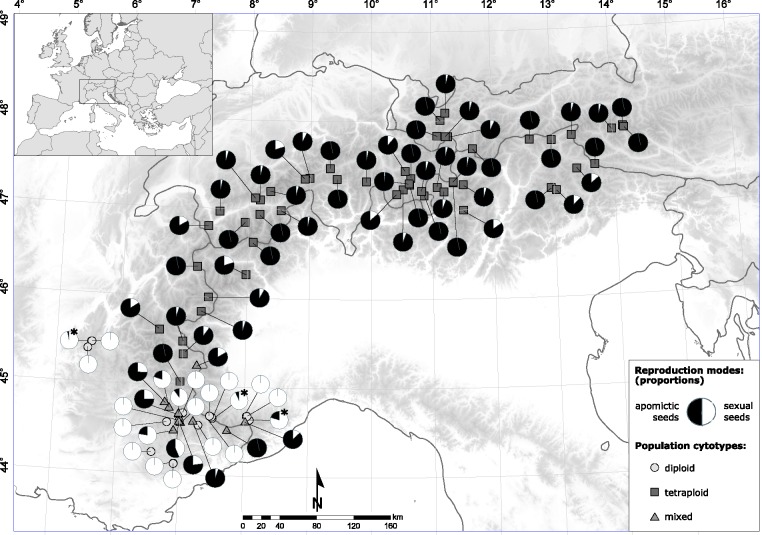
Geographical distribution of *Ranunculus kuepferi* reproduction modes of analyzed populations on a map of the European Alps with elevation model. Pie diagrams indicate proportions of sexual (white) and apomictic seed (black) formation within the populations, diploids with apomicts are marked by an asterisk. Cytotypes: 2×, diploid populations; 4×, tetraploid populations; mixed, populations consisting of two or more different cytotypes, including triploids.

A total of 551 individuals from 81 populations provided sufficient material for analyses. Flow cytometry of leaves revealed 132 diploid, 25 triploid and 394 tetraploid individuals. Most populations were of uniform cytotype: we discovered 18 diploid (22.2 %) and 52 (64.2 %) tetraploid populations, while 11 (13.6 %) populations had a mixed composition of different cytotypes ranging from di- to even pentaploid plants. Triploid individuals were found scattered across mixed populations in the contact zone of diploids and tetraploids (Fig. 1), but did not constitute any single uniformly triploid population.

Surprisingly, flow cytometric seed screening revealed that only a minority of 34 (42.0 %) populations reproduced uniformly (obligate sexual or apomictic seed formation), while 47 populations (58.0 %) had a mixed mode of reproduction (i.e. both sexual and apomictic seed formation). We excluded seeds from three pentaploid and one hexaploid individuals from further analysis. Seeds formed via partial apomixis (BIII hybrids, see below), were analyzed but also excluded from further statistical analysis.

### Modes of seed formation

The analysis of a total of 2795 seeds revealed in general a noticeably high variability in seed formation among all plants studied (Table 1). In diploids, sexuality was by far the most prevalent mode of reproduction. The majority of seeds (94.7 % from 700 in total) were derived sexually. In each case, we observed embryo to endosperm ratios of 2C:3C, which is characteristic for sexual development. Normal fertilization with a reduced pollen results in a fertilized diploid embryo (1Cm + 1Cp) and a triploid endosperm formed by the fusion of two reduced maternal central nuclei subsequently fertilized with a second pollen sperm nucleus (1Cm + 1Cm + 1Cp; pathway A in Table 1; **[see Supporting Information—Fig. S1a]**). Nonetheless, a tiny fraction of 4.9 % (34) of diploid seeds originated via apomixis (pathways A2–A4 in Table 1; **[see Supporting Information—Fig. S1b]**). Autonomous endosperm development in diploids (2Cm + 2Cm) was not observed. Instead, we recorded three cases (0.4 %) of ploidy shifts in the embryo among diploid apomictic seeds, originating from fertilization of unreduced egg cells (pathway AB). Although rather rare, principally all cytotypes were prone to that, resulting in the formation of so-called BIII-hybrids (Nogler, 1984), which exhibit partial apomixis, i.e. a combination of apomeiosis and fertilization. Due to the availability of maternal leaf ploidy data for all plants, BIII hybrids (embryos with higher ploidy than the mother plant) could be determined with great certainty.

Tetraploid plants also exhibited a profound level of variance in mode of reproduction. While their primary reproduction mode was conversely based on apomictic pathways that account for 91.2 % (1797) of the yielded seeds (pathways C2–D4), a total of 6.0 % (118) were developed sexually (pathway C; **[see Supporting Information—Fig. S1c]**). Beside some ambiguous cases, apomictic seeds could be assigned to nine different developmental pathways (Table 1). With 99.2 % (1782), the vast majority of apomictic seeds were formed pseudogamously. Unreduced pollen should result in highly polyploid endosperm (12C or 16C), depending on whether one or both sperm nuclei were used (pathways C3 or C5 respectively; **[see Supporting Information—Fig. S1d–f]**), while reduced pollen can fertilize the endosperm with either one (10C) or two (12C) of its sperm nuclei. Alternatively, in some cases also trinucleate endosperm can explain the pattern (Talent and Dickinson, 2007, see Table 1). A total amount of 16 seeds (0.8 %) exhibited an octoploid endosperm, indicating autonomous formation from unreduced central cells (8C); pathway C6. BIII hybrids with a shift to higher ploidy levels in the embryo were discovered in 43 seeds (2.2 %); pathway CB. In 21 cases (1.1 %), the pathways could not be reconstructed.

In triploid plants, 119 seeds were produced by apomictic pathways (95.2 %; pathways B2–D1), only six (4.8 %) were derived sexually. Within the apomictic pathways, two seeds were found with a diploid embryo, while three seeds contained tetraploid embryos. Autonomous endosperm formation was not observed.

### Variance and geographical pattern of reproduction mode of populations

Based on the individually pooled FCSS data, all plants could be assigned to three distinct groups. Most individuals (450, 81.7  %) reproduce uniformly, being either obligate sexual or obligate apomictic. The remaining 101 individuals (18.3 %) are facultatively apomictic (= mixed). In the 132 diploids, beside the typically obligate sexual plants (95.4  %), we found six individuals (4.6  %) being facultative apomictic, but no individual with obligate apomixis. The facultative apomictic diploids appeared in three geographically isolated populations (Fig. 1). Tetraploids were predominantly obligate apomictic, 90 plants (22.8 %) exhibited facultative apomixis to varying degrees, but none of the tetraploid individuals was obligate sexual. In triploids, five individuals (20.0  %) were discovered being facultative sexuals, while their main reproductive mode is based on obligate asexual pathways, like in tetraploids.

Among diploid populations, 15 were found to be obligate sexual (88.9  %) whereas three contained individuals with a mixed formation of seeds. In contrast, tetraploids exhibited 34 (65.4  %), a significantly greater proportion of populations with facultative sexuality [*t*(70) = 4.5, *P *< 0.01]. Only 18 populations were found to be obligate apomictic. Facultative apomixis therefore exhibits a significant correlation to ploidy [*F*(2, 78) = 379.7, *P *< 0.01]. The 10 populations comprising varied cytotypes all express a mixed mode of reproduction. Overall, the occurrence of facultative sexuality is evenly scattered across populations and spread over the entire Alps in a seemingly random matter (Fig. 1). The Mantel test did not indicate a correlation of mode of reproduction and geographical distance (*Observation* = 0.016; *P* = 0.17).

### Seed set and morphological fitness parameters

Variation in seed production of *R. kuepferi* was remarkably high among all three cytotypes. The highest variance was found in diploids with values ranging from 7.2 % to 96.7 % fully developed seeds. Within tetraploids, values varied between 4.1 and 83.3 %. On average, tetraploids produced 30.8 % ± 13.4 well-developed seeds (∼15 seeds: plant) and therefore showed a significantly lower proportion [*t*(522) = −9.6, *P* < 0.01] than diploids (46.8 % ± 24.1, ∼20 seeds: plant). Triploids showed a lesser variance (5.8–50.8  %) but the lowest mean (25.8 % ± 10.4) of all three groups. Hence, triploids differed significantly from diploids [*t*(154) = −5.8, *P* < 0.01] but not from tetraploids [*t*(418) = −1.5, *P* = 0.29]. Focusing on the reproduction mode, obligate sexual individuals (mean 47.0 % ± 24.1) had a significantly higher seed set than obligate apomicts [*t*(446) = 9.3, *P* < 0.01] and facultative sexual individuals [*t*(224) = 7.3, *P* < 0.01].

Analysis of data at the population level revealed similar results: uniformly diploid populations had the highest seed set (mean 41.9 % ± 17.6), uniformly tetraploid populations produced significantly [*t*(70) = −4.5, *P* < 0.01] fewer filled achenes (mean 27.1 % ± 9.2) and mixed cytotype populations performed worst (mean 23.4 % ± 10.9), as they comprised plants of tetra- and higher ploidies beside triploids and only a few diploid samples.

**Figure 2:**
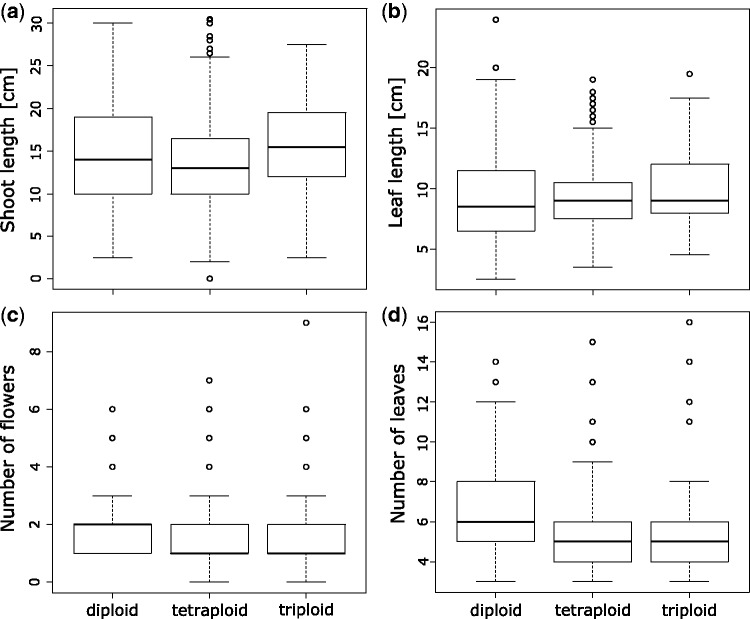
Boxplots of field collected fitness parameters on individual level of *Ranunculus kuepferi* cytotypes. Outliers are presented as black circles (o). (**a**) Total length of main shoots from ground to flower base, respectively fruit base. (**b**) Length of the longest leaf per plant. (**c**) Total number of flowers, respectively buds or fruits, per plant. (**d**) Total number of ground and shoot leaves per plant.

Morphometric data showed that diploid individuals tended to be taller than tetraploids [*t*(1044) = 2.8, *P* < 0.01; Fig. 2a]. Leaf length did not differ significantly between cytotypes [*F*(2, 1125) = 2.5, *P* < 0.07], with diploids and tetraploids being particularly similar [*t*(1043) = 0.3, *P* = 0.94]; Fig. 2b. Diploids developed significantly more flowers [*t*(1045) = 3.7, *P* < 0.01, Fig. 2c] and more leaves [*t*(1045) = 9.3, *P* < 0.01, Fig. 2d] than tetraploids (Fig. 2c). Moreover, for each of the two factors a negative correlation to elevation was found in diploids [*r_s_*(81) = −0.15, *P* = 0.01 and *r_s_*(81) = −0.13, *P* = 0.03]. Triploids had an intermediate phenotype with significantly more leaves [*t*(872) = 3.1, *P* < 0.01] and flowers [*t*(872) = 1.9, *P* < 0.01] than tetraploids, but less leaves [*t*(343) = −2.5, *P* = 0.03] and flowers [*t*(343) = −0.4, *P* = 0.91] than diploids.

### Ecological factors

**Figure 3:**
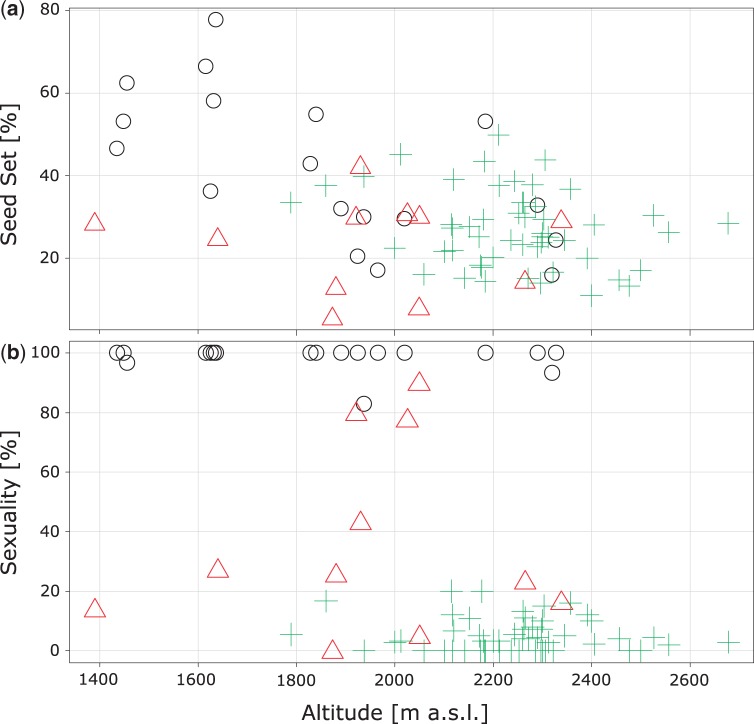
Scatterplots showing variation between *Ranunculus kuepferi* cytotypes on population level regarding reproduction parameters in dependence of elevation. Black circle (o): diploid populations; green cross (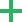): tetraploid populations; red triangle (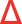): population consisting of individuals with different cytotypes (mixed). (**a–b**) Proportions of well-developed achenes per flower and plant (seed set, **a**), as well as proportions of facultative sexuality contributing to seed development (**b**) in dependence of elevation above sea level (elevation).

Elevation of sites was significantly positively correlated to ploidy [*r_s_*(81) = −0.56, *P* < 0.01]. Tetraploid populations predominantly occurred at higher elevations (mean 2243.9 m a.s.l. ± 70.6) than diploid (mean 1855.6 m a.s.l. ± 245.7) and mixed ones (mean 1942.6 m a.s.l. ± 251.7) (Fig. 3). Regarding reproductive fitness, diploids exhibited a strong negative correlation of seed set to elevation [*r_s_*(18) = −0.68, *P < *0.01], while tetraploid and mixed populations did not. The amount of facultative sexuality showed to be correlated to ploidy [*r_s_*(81) = −0.45, *P < *0.01].

**Figure 4:**
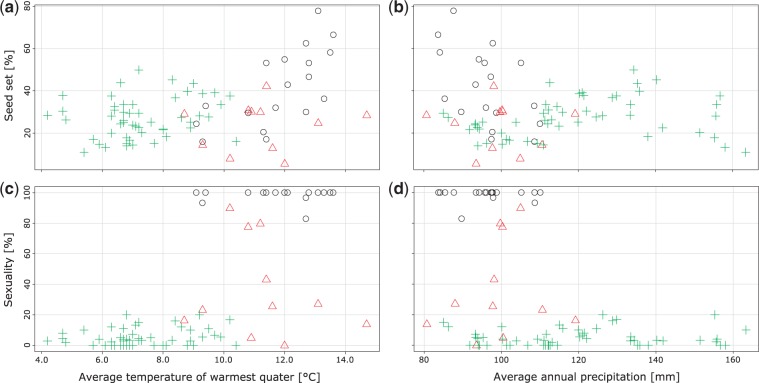
Scatterplots of ecological against developmental parameters on population level of *Ranunculus kuepferi* differentiated by cytotype. Black circle (o): diploid populations; green cross (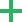): tetraploid populations; red triangle (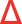): population consisting of individuals with different cytotypes. (**a–b**) Proportions of well-developed achenes averaged per flower and plant (seed set) in dependence of average temperatures of the three warmest months (**a**; BIO10, WorldClim) as well as in dependence of the average annual precipitation (**b**; BIO12, WorldClim). (**c–d**) Facultative sexuality in dependence of average temperatures (**c**) and in dependence of precipitation (**d**).

However, the temperature of the warmest quarter is closely negatively correlated to elevation [*r_s_*(81) = −0.85, *P < *0.01] and hence similarly to seed set [*r_s_*(81) = 0.33, *P < *0.01] and reproduction mode [*r_s_*(81) = −0.68, *P < *0.01]. Average mean temperatures of the warmest quarter at sites were lowest among tetraploids (7.3 °C ± 0.9), but did not differ significantly between diploid (11.8 °C ± 1.4) and mixed populations (11.3 °C ± 1.6) (Fig. 4a and c).

Precipitation was significantly negatively correlated to proportion of facultative sexual individuals within a population [*r_s_*(81) = −0.40, *P < *0.01], and was highest at the sites of tetraploids (mean 119.1 mm ± 18.0). Here too, diploid (mean 96.2 mm ± 8.6) and mixed populations (mean 99.4 mm ± 9.8) did not differ significantly from each other. Seed set was not related to this variable [*r_s_*(81)* *= 0.004, *P = *0.97] (Fig. 4b and d). Precipitation was positively correlated with ploidy [*r_s_*(81) = −0.48, *P < *0.01].

## Discussion

In this study, we present the first quantitative study of modes of seed formation in *R. kuepferi* over the entire distribution range in the European Alps. Additional to the geographical differentiation of cytotypes recorded previously (Cosendai and Hörandl, 2010), we were able to demonstrate a positive correlation of apomictic mode of reproduction to elevation and related environmental factors. On average, apomictic tetraploids are found at higher elevations than sexual diploids. Hence, the niche shift to colder temperatures as reported by Kirchheimer *et al.* (2016) is mostly an effect of elevation (average decrease of 0.6 °C per 100 m; Nagy and Grabherr 2009). If the shift to colder temperature would be just a latitude effect (average decrease of temperature c. 0.75 °C per degree), then the northernmost populations would have to occur at lower elevations (c. 300 m) to keep their niche constant (e.g. many high alpine plants occur in the Arctic on sea level; Körner, 2003). However, this is not the case in *R. kuepferi*. This niche shift to higher elevations would be also in line with the general trend of apomicts to occur in more extreme habitats compared to their diploid sexual progenitors, as postulated by the Frozen Niche Variation model. However, there is no niche expansion, as the amplitude was not expanded to lower elevations.

Large-scale studies of well-known examples that exhibit diverging distributions between sexuals and asexuals, such as the *Crepis* complex (Babcock and Stebbins, 1938; Bierzychudek, 1985), *Paspalum simplex* (Urbani *et al.*, 2002), *Taraxacum officinale* agg. (Van Dijk, 2003) and *Crataegus* (Lo *et al.*, 2013) indicate classical patterns of geographical parthenogenesis, with polyploid apomicts occupying larger and colder areas than their diploid sexual relatives. Other examples in which apomicts do not possess an unequal distribution or even a smaller one compared to sexuals are *Hieracium pilosella* (Fehrer *et al.*, 2007) and particularly the *Boechera holboellii* complex, with apomicts occurring more in southern parts of North America (Dobeš et al., 2004a, b) and an overall diffuse distribution pattern (Sharbel *et al.*, 2005; Mau *et al.*, 2015). However, in the latter two cases, the ploidy differentiation between modes of reproduction is not so pronounced, as sexuals in *Hieracium pilosella* are tetraploids (Mráz et al., 2009), while apomicts in *Boechera* include triploid and diploid taxa (Aliyu *et al.*, 2010; Lovell *et al*., 2013). Hence, only a combination of polyploidy and asexuality versus diploid sexuality seems to establish a pattern of geographical parthenogenesis in plants (Hörandl, 2006; Hörandl *et al*., 2008).

We found a surprisingly high diversity in the reproductive pathways in *R. kuepferi*, both on individual and on population level. A remarkably high proportion of tetraploid individuals formed seeds which are, to a varying degree, based on sexual pathways. Facultative apomixis is quite typical for plants reproducing via apospory because apomictic and sexual development run initially in parallel (Asker and Jerling, 1992; Krahulcová *et al*., 2004; Dobeš *et al.*, 2013; Klatt *et al.*, 2016). Apospory was observed in *R. kuepferi* by Burnier et al. (2009). Generally, the mode of reproduction in *R. kuepferi* seems to be even more variable than expected. Indeed, most of the older studies on asexual reproduction in plants suggest that apomixis and polyploidy are almost exclusively and functionally correlated (Gustafsson, 1946; Bierzychudek, 1985; Carman, 1997; Koltunow and Grossniklaus, 2003). In contrast, our results provide evidence that this is not necessarily the case. Although most diploid individuals reproduce strictly sexual, a non-negligible amount of plants from three independent, geographically separated populations feature facultative apomixis (Fig. 1). Across numerous studied taxa, diploid plants expressing regularly functional apomictic seed formation in natural diploid populations are only known in the genus *Boechera* (Dobeš *et al.*, 2006; Aliyu *et al.*, 2010) and in various *Paspalum* species (Siena *et al.*, 2008). *Boechera* has however a very complex taxonomic structure with more than 80 highly polymorphic species and hybrids (Koch *et al.*, 2003; Dobeš et al., 2004a, b). Several studies found high levels of heterozygosity in diploid apomictic species of the complex, concluding them to be of hybrid origin (Beck *et al.*, 2012; Dobeš et al., 2004a, b; Kiefer *et al.*, 2009; Kiefer and Koch, 2012; Schranz *et al.*, 2005). A hybridogeneous origin as prerequisite for diploid apomixis cannot be completely ruled out in *Boechera*. In *Ranunculus auricomus*, diploid apomicts were observed just in experimentally produced dihaploid progenies, or in synthetic hybrids under garden conditions (Nogler, 1984; Hojsgaard *et al.*, 2014). Our quantitatively orientated study illustrates that large scale seed screenings are required to detect small but non-negligible amounts of facultative apomixis in diploids in natural populations, which would otherwise remain overlooked.

We found most asexually reproducing individuals at higher elevations under generally colder conditions. However, we cannot disentangle this phenomenon from ploidy effects as observed by Kirchheimer *et al.* (2016). Even among tetraploid populations at highest elevations, both sexual and apomictic seed formation occurs (Fig. 3b). A non-linear correlation of apomixis and elevation was also observed in subnivale plants in the highest zones of the Alps where sexuality is prevalent (Hörandl *et al.*, 2011). The same phenomenon appears in high latitudes, as apomicts become rare in arctic and subantarctic floras (Asker and Jerling, 1992). Furthermore, there is no geographical pattern in the degree of facultative sexuality over the whole distribution range of tetraploids (Fig. 1). The lack of a correlation of geographical distance to frequencies of apomictic reproduction does not support the idea of a prevalence of long distance dispersal for founding apomictic populations. Instead, we assume predominantly a fast stepwise dispersal aided by self-fertility and apomixis for the colonization of the Alps. This scenario is also supported by the lack of population genetic structure within the tetraploid range (Cosendai *et al.*, 2013). Diploid populations with apomictic seed formation occur just in the diploid refugial areas, implying that apomixis alone is unsuccessful (Fig. 1). Tetraploid obligate sexual individuals were not observed in our study. Taken together, neither apomixis nor polyploidy alone explain the geographical pattern; only the combination of both factors is successful.

Focusing on the reproductive fitness of *R. kuepferi*, we found no positive effect of ploidy on the development of seeds. Seed set is overall significantly lower in polyploids than in diploids. However, diploids showed a significant negative correlation to elevation, and were in the highest populations between 2200 and 2400 m within the range of seed set of tetraploids (Fig. 3a). Seed set in diploids may be in highest elevations negatively influenced by pollinator limitation, or by a worse adaptation of reproductive tissues to cold temperatures (see below). Tetraploids, in contrast, remain more stable on a low level of seed set over the whole altitudinal gradient (Fig. 3a). Low seed set is probably a consequence of autopolyploidy. In sexual autopolyploids, profound difficulties may arise during chromosome segregation at meiosis due to the increased complexity of pairings among doubled and therefore identical chromosome sets, which mostly pair as multivalents. An efficient segregation of such multivalents in case of autotetraploid *R. kuepferi* remains difficult (Cosendai *et al.*, 2011). Common segregation issues within enlarged chromosome sets can lead to disadvantageous irregularities such as aneuploidy (Birchler *et al.*, 2007; Ozias-Akins and Van Dijk, 2007). In polyploid *R. kuepferi*, we have discovered only very few cases of triploid embryos in the seed screening. We suppose that triploid megaspores resulting from disturbed meiosis largely abort during gametogenesis, which would explain the high proportions of aborted seeds (see also Cosendai and Hörandl, 2010). High proportions of aborted pollen (Huber, 1988) support this hypothesis. However, these developmental disturbances appear to be independent from elevation and associated environmental factors (Fig. 3a).

Nevertheless, despite a significantly worse overall reproductive fitness, tetraploid apomicts seem to be the only successful cytotype occupying the Alps as evident from the observed distribution patterns. One could argue this might result from implications of polyploidization itself. There is a long-lasting debate about the advantages or disadvantages of polyploidy and many hints are yet available which suggest a quite beneficial effect of genome doubling under certain circumstances (Ohno, 1970; Comai, 2005; Otto, 2007; Soltis and Soltis, 2009). Especially if polyploids manage to adapt, they possess the potential to establish efficient competitors to their diploid ancestors (Comai, 2005). A multitude of studies suggest that possible success is mainly based on a potential manifold advantage of polyploidy. In polyploids, overall performance is often increased due to the heterosis effect (Birchler *et al.*, 2010). According to some studies, putative autopolyploids even show stronger heterosis compared to allopolyploid hybrids (Bingham *et al.*, 1994; Birchler *et al.*, 2010).

However, the results of our morphological measurements in *R. kuepferi* were the contrary to expectations of heterosis effects: diploids proved to produce more leaves and flowers, exhibiting a seemingly higher vigour at least compared to the tetraploid plants, with a negative correlation to elevation. But only tetraploids occurred at higher elevations >2400 m.a.s.l., where the growing season starts later and a shorter vegetation period is available for growth. Moreover, low temperatures slow down cell cycle and cell differentiation processes (Körner, 2003). In general, alpine plants produce fewer cells with a tendency to keep cell size constant, and as a result alpine plants often show a syndrome of ‘alpine dwarfism’ which is also adaptive to harsh climatic conditions at higher elevations (Körner, 2003). Another potential limiting factor for successful plant reproduction is summer frost at high elevations, especially when reproductive tissues are not covered by snow and exposed to frost (Ladinig *et al.*, 2013). In situations of summer frost, the taller growth and richer flower production of diploid *R. kuepferi* could be rather a disadvantage for successful seed production. Hence, tetraploid *Ranunculus kuepferi* probably adjusts its growth better to high alpine conditions. To which extent the observed morphological parameters are influenced by phenotypic plasticity or represent a heritable feature, needs to be studied.

Assets and drawbacks related to performance are not limited to hybrid vigour. Performance can also be positively influenced by direct consequences of present genome duplication. Polyploids maintain heterozygosity which can be important when isolated and severely bottlenecked, and autopolyploids preserve heterozygosity even better than hybrids (Comai, 2005). In *R. kuepferi*, genetic diversity in tetraploid populations was as high as in diploids in all measures (Cosendai *et al.*, 2013). Preserving genetic diversity and heterozygosity could have been helpful to escape the consequences resulting from inbreeding depression and genetic bottlenecks, as tetraploid apomictic individuals were spreading rapidly eastwards from the original core area in the south-western Alps.

Furthermore, gene duplication establishes the ability to diversify gene functions (Prince and Pickett, 2002; Adams and Wendel, 2005; Moore and Purugganan, 2005). Sub- or even neofunctionalization of redundant genes possibly cause niche shifts (Lynch and Walsh, 2007). Additionally, gene expression may be modified by the occurrence of doubled alleles (Wang *et al.*, 2004; Hegarty *et al.*, 2006, 2011). One should assume that expression patterns of most diploid sexual genotypes in a population are optimized to certain environmental conditions on site (Comai, 2005). However, relevant differences in effects on transcriptomes were detected among allopolyploidy and mere genome doubling (Hegarty *et al.*, 2005, 2006). Hegarty *et al.* (2006) further suggested that genome doubling has a ‘calming effect’ on hybridization-induced transcriptome shock. Thus, in autopolyploids, regulatory changes need not necessarily be unfavourable, but might allow for faster adaptation.

We hypothesize that a physiological ability to withstand conditions at high elevations is positively influenced by polyploidization in *R. kuepferi*. Aside from ranges, absolute elevations of tetraploid populations are significantly higher than in diploids. It is known that cell structures fundamentally change with the doubling of the genome, almost always resulting in an enlargement of cell size (Melaragno *et al.*, 1993; Levin, 2002). Compared to its alpine competitors, *R. kuepferi* is a fast developing plant, which is among the first sprouting and flowering after snow melting; after fruiting, the vegetative parts wither and disappear rapidly. Diploids generally bloom, fruit and ripe earlier than the tetraploids. Apart from climatic differences influencing growing seasons due to altitudinal ranges in the wild, this tendency is also apparent in cultivation when kept under same conditions (C. Schinkel and E. Hörandl, pers. obs.). An increase in cell size and a concordantly altered surface to volume ratio are known to be advantageous for cells with high metabolic rates (Comai, 2005), and may allow a reduction of cell number (see above). Furthermore, alpine plants have to adapt their carbon uptake to lower CO_2_ partial atmospheric pressure (Körner, 2003). Taken together, the observed tendency to reduce growth in tetraploid *R. kuepferi* is probably an adaptation to the short vegetation period, to lower temperatures, and to lower carbon availability at higher elevations. Polyploidy has further pronounced effects on photosynthesis performance, e.g. increasing electron transport capacity (Coate *et al.*, 2012), which might be advantageous under higher UV irradiation and light intensity in higher elevations. Other aspects of the observed niche shift of tetraploids, like a tendency to more acid soils (Kirchheimer *et al.*, 2016), could also relate to polyploidy rather than to mode of reproduction.

Apomictic development, in turn, may help to accelerate seed formation under conditions of shorter vegetation periods. Diploids apparently lost their fitness advantage in higher elevations. We could not observe differences in timing of female gametophyte development between sexual and apomictic pathways (C. Schinkel, unpubl. data); but, embryogenesis and seed development could be accelerated, as cross-fertilization and pollinator visits are not needed for tetraploids. Previous experimental work has shown that tetraploids are self-fertile whereas diploids are largely self-incompatible (Cosendai *et al.*, 2013). Hence, reduced seed set in diploid sexuals at highest elevations could be due to pollinator limitation (Arroyo *et al.*, 1982; Körner, 2003). In contrast, apomictic colonizers do not experience pollinator and mate limitation, which are important aspects of Baker’s law (Pannell *et al.*, 2015).

In this study, we primarily focused on colder temperatures which prevail at higher elevations and may directly influence reproduction. De Storme *et al.* (2012) found that short periods of cold stress are able to induce the production of a certain amount of polyploid pollen in diploid *Arabidopsis thaliana*. According to our FCSS data, several asexual reproduction pathways could theoretically involve unreduced pollen, mainly found in tetraploids (Table 1). However, the male contribution to endosperm DNA content by unreduced pollen overlaps in most pathways with that of a possible double fertilization of the endosperm with both sperm nuclei of reduced pollen. Based only on the data obtained by the FCSS, one can only ascertain a few cases of unreduced pollen (pathway B5, Table 1). On the female side, unreduced egg cells were formed in all apomictic pathways and in BIII hybrids, and occurred in all cytotypes. Experimental cold treatments triggered spontaneous apomixis in diploids, suggesting a direct influence of temperature on female gamete formation (S. Klatt, unpubl. data). Whether all of these cases are due to the development of somatic cells (apospory) as reported by Burnier *et al.* (2009) or also to unreduced megaspores formed by restitutional meiosis (diplospory), needs to be studied. Moreover, we analyzed wild populations and used climate data of relatively low resolution. However, environmental parameters in the Alps may change on small scales (Kirchheimer *et al.* 2016). Thus, obtained results do not account for any microclimatic variation among sample sites. Whether there is a direct influence of cold on polyploidization in *R. kuepferi* or not, remains an open question at present to be addressed.

## Conclusions

In summary, the major outcome of this study underpins the strong correlation of ploidy with elevation and correlated climatic variables. Conversely, the mode of reproduction turned out to be of substantial variance independent of cytotypes and indeed is also correlated to elevation, but cannot be disentangled from polyploidy. A combination effect, postulated by Hörandl (2006), likely applies to *R. kuepferi*. Positive effects of polyploidy are probably morphological and physiological adaptations to conditions at high elevations, enabling them to conduct a significant niche shift in the allopatric range (Kirchheimer *et al.* 2016). Positive effects of apomixis are probably increasing the capacity for a rapid range expansion by founding populations with single diaspores without mate and pollinator limitation following Baker’s law (Pannell *et al.*, 2015). In higher elevations, apomixis may help to shorten developmental pathways to seed formation which is an advantage in short vegetation periods. Facultative sexuality, in turn, preserves genetic diversity and adaptive potential in small founder populations (Cosendai *et al.*, 2013).

## Sources of Funding

The work was supported by the German Science Foundation “Deutsche Forschungsgemeinschaft DFG” [grant number HO 4395/1-1] to [EH] and the Austrian Science Fund FWF [grant number I 1189] to [SD].

## Contributions by the Authors

All authors were considerably involved in the organization of the field trips as well as the collection of plants. Christoph Carl-Friedrich Schinkel is the main author of the article. He conducted the flow cytometry analysis, microscopic studies (mentioned in the discussion section), seed set counting, main parts of the statistical analysis and large part of the text. Bernhard Kirchheimer, Agnes Dellinger, Manuela Winkler and Stefan Dullinger provided results of morphology and helped with statistics. Simone Klatt helped with flow cytometry and microscopy. Elvira Hörandl helped organizing, developing theories and provided substantial knowledge. All authors were involved in writing this article, providing insightful ideas, constructive critics and text passages to a certain degree.

## Conflict of Interest Statement

None declared.

## Supplementary Material

Supplementary Data

## Supporting Information

The following additional information is available in the online version of this article —

 **Figure S1**. Flow cytometry histograms of the most frequent pathways.

 **Figure S2**. Boxplots of seed set between *Ranunculus kuepferi* cytotypes.

 **Table S1**. Table of sampled populations with geographical reference.
