# Supplementary material for: Correlations of polyploidy and apomixis with elevation and associated environmental gradients in an alpine plant
Source: AoB Plants. 2016 Oct 26;8:plw064. doi: 10.1093/aobpla/plw064 (PMC5091893; doi:10.1093/aobpla/plw064)
Supplement: Supplementary Data [file supp_8_plw064_index.html]

Correlations of polyploidy and apomixis with elevation and associated environmental gradients in an alpine plant — Supplementary Data 

# Correlations of polyploidy and apomixis with elevation and associated environmental gradients in an alpine plant

## Supplementary Data

files

- Supplementary Data - doc file
