# Supplementary material for: Correlations of polyploidy and apomixis with elevation and associated environmental gradients in an alpine plant
Source: AoB Plants. 2016 Oct 26;8:plw064. doi: 10.1093/aobpla/plw064 (PMC5091893; doi:10.1093/aobpla/plw064)
Supplement: Supplementary Data [file supp_plw064_aobplants-16055-T-s01.doc]

**SUPPORTING INFORMATION**


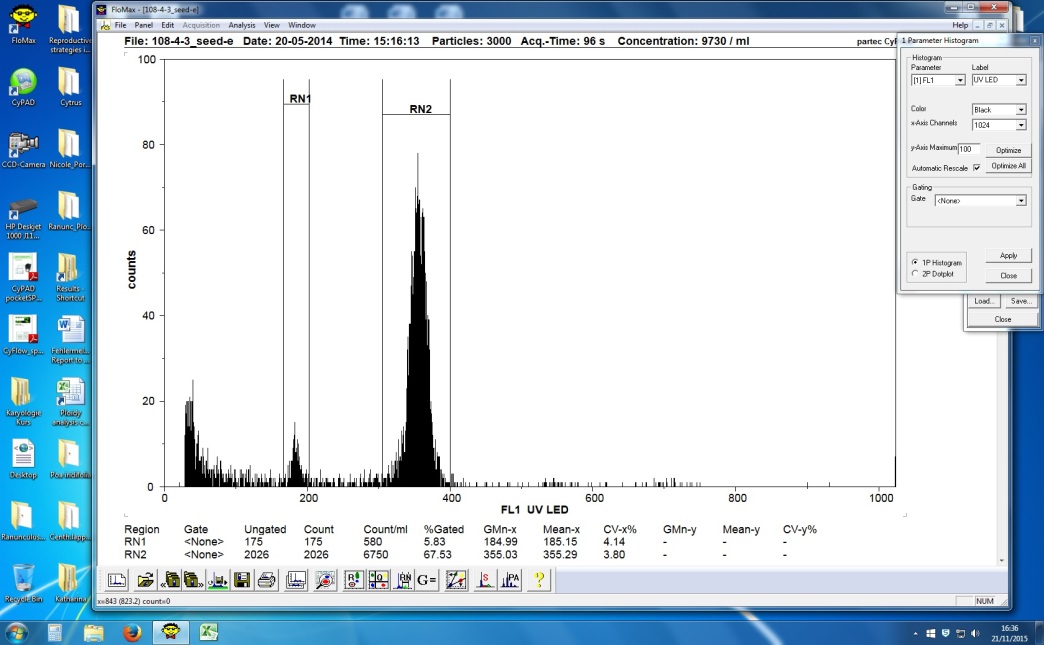


0

100

200

300

400

500

600

800

1000

RN1

RN2

f

0

100

200

300

400

500

600

800

1000

0

20

40

60

80

100


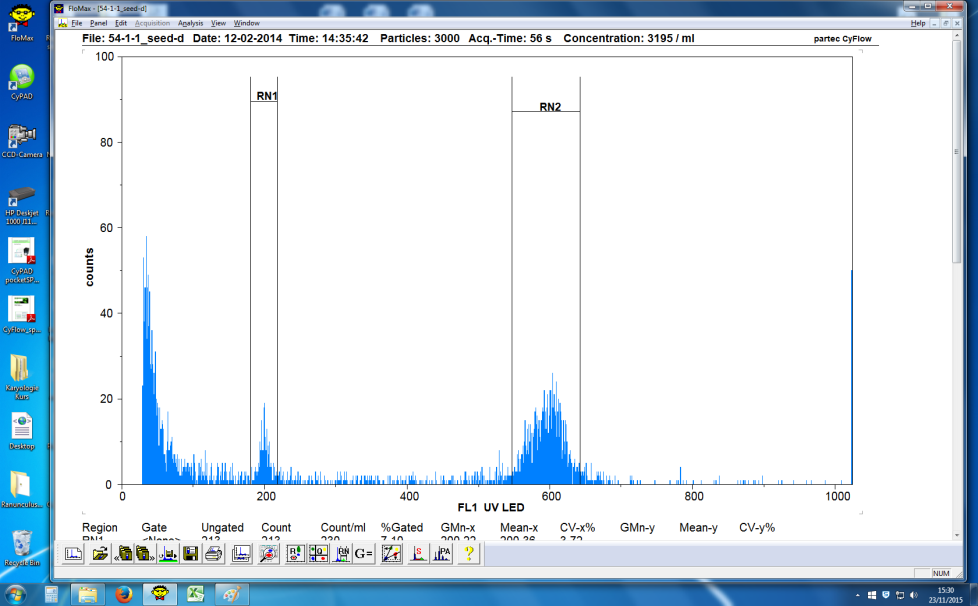


RN1

RN2

G2

e


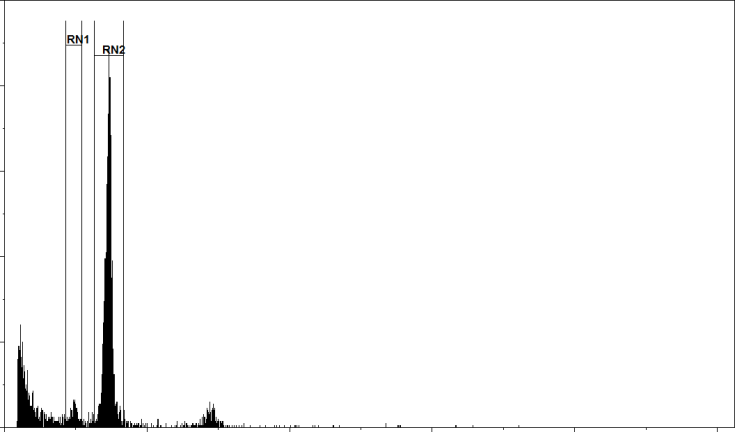


0

20

40

60

80

100

RN1

RN2

a

G2


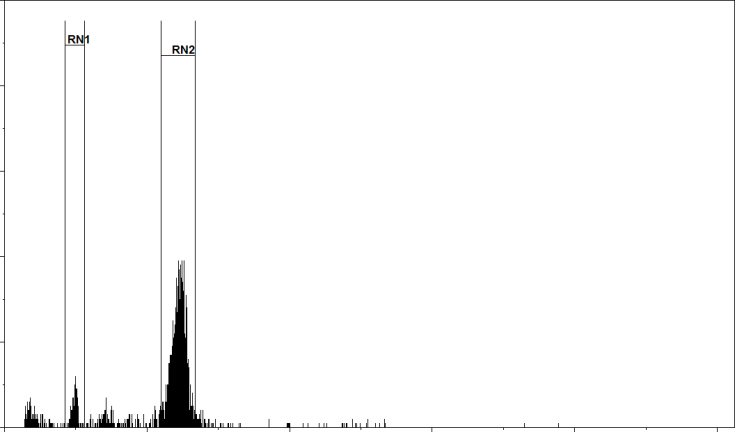


RN1

RN2

b

G2


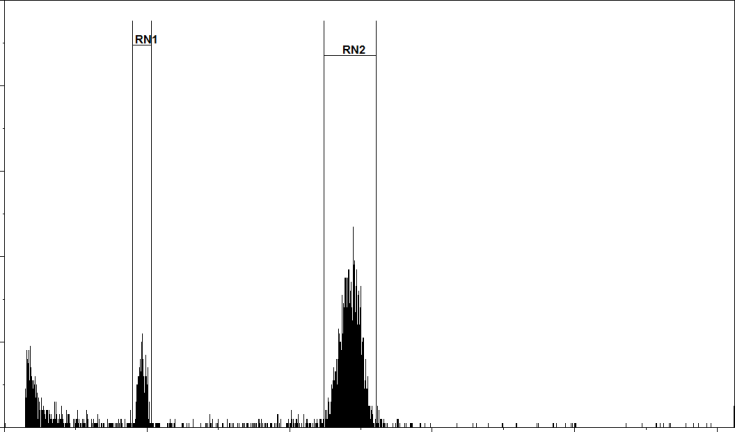


RN1

RN2

d

G2


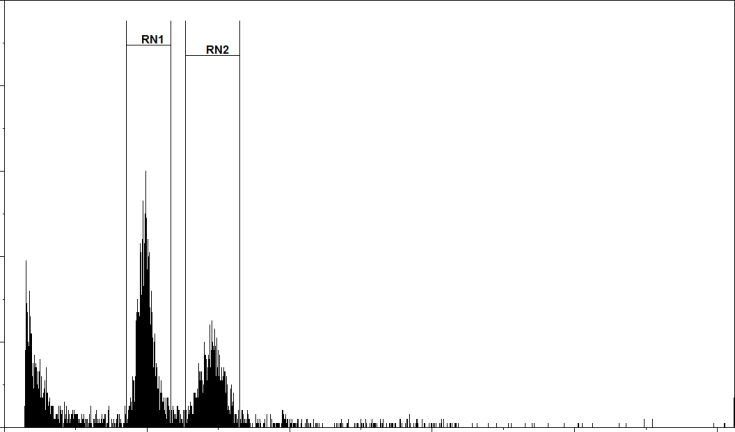


0

20

40

60

80

100

RN1

RN2

c

G2

**Figure S1.** Flow histograms of the most frequent seed formation pathways inferred from FCSS (see Table 1). RN1, embryo peak; RN2, endosperm peak; G2, G_2_-phase of cell cycle. **a:** Sexual seed formation in diploids (pathway A); **b:** Apomictic seed formation in diploids, pseudogamous endosperm (one reduced sperm nucleus; pathway A2); **c:** Sexual seed formation in tetraploids (pathway C); **d:** Apomictic seed formation in tetraploids, pseudogamous endosperm (one reduced sperm nucleus; pathway C2); **e:** Apomictic seed formation in tetraploids, pseudogamous endosperm (either two reduced sperm nuclei or one unreduced sperm nucleus; pathway C3); **f:** Apomictic seed formation in tetraploids, autonomous endosperm development (pathway C6).

**Figure S2.** Boxplots of seed set between *Ranunculus kuepferi* cytotypes, based on percentage of well-developed achenes per flower, averaged per plant. Outliers are presented as black circles (o).

**Table S1** Table of sampled populations with geographical reference. pop = population number; A = Austria, CH = Switzerland, F = France, I = Italy, north and east denote coordinates; alt = altitude in m a.s.l. Voucher specimens have been deposited in the herbarium of the University of Göttingen (GOET).

| **pop** | **country** | **region** | **north** | **east** | **alt** |
| --- | --- | --- | --- | --- | --- |
| 1 | F | Provence-Alpes-Côte d'Azur | 43.99972 | 7.43028 | 2057 |
| 2 | F | Provence-Alpes-Côte d'Azur | 44.09583 | 7.35950 | 1873 |
| 3 | F | Provence-Alpes-Côte d'Azur | 44.20028 | 7.15639 | 2291 |
| 4 | F | Provence-Alpes-Côte d’Azur | 44.17528 | 6.89806 | 2050 |
| 13 | F | Rhônes-Alpes | 44.54694 | 6.70361 | 2059 |
| 14 | F | Provence-Alpes-Côte d'Azur | 44.35489 | 6.50558 | 1880 |
| 16 | F | Provence-Alpes-Côte d'Azur | 45.03483 | 6.40242 | 2078 |
| 17 | F | Provence-Alpes-Côte d'Azur | 45.05069 | 6.39078 | 2357 |
| 20 | CH | Valais | 46.34719 | 7.72411 | 2200 |
| 23 | F | Provence-Alpes-Côte d'Azur | 43.74931 | 6.65744 | 1616 |
| 24 | F | Provence-Alpes-Côte d'Azur | 44.15083 | 6.54361 | 1925 |
| 25 | F | Provence-Alpes-Côte d'Azur | 44.90128 | 5.47617 | 1435 |
| 26 | F | Provence-Alpes-Côte d'Azur | 44.90033 | 5.46900 | 1456 |
| 27 | F | Rhônes-Alpes | 44.83933 | 5.42422 | 1449 |
| 28 | I | Piemonte | 44.24414 | 7.62906 | 1685 |
| 29 | I | Piemonte | 44.23111 | 7.61722 | 2020 |
| 30 | I | Piemonte | 44.18458 | 7.62700 | 1743 |
| 31 | I | Piemonte | 44.19522 | 7.65922 | 1937 |
| 32 | I | Piemonte | 44.20667 | 7.14750 | 2320 |
| 33 | I | Piemonte | 44.21305 | 7.14667 | 2328 |
| 34 | F | Rhônes-Alpes | 45.24244 | 6.95189 | 2120 |
| 36 | F | Rhônes-Alpes | 45.38542 | 7.04372 | 2152 |
| 37 | I | Val d'Aosta | 45.61622 | 7.55261 | 2115 |
| 38 | F | Rhônes-Alpes | 45.67822 | 6.87800 | 2182 |
| 40 | CH | Valais | 46.08250 | 7.01250 | 1860 |
| 41 | I | Val d'Aosta | 45.93195 | 7.63944 | 2174 |
| 42 | CH | Valais | 46.43300 | 7.86492 | 1789 |
| 43 | CH | Valais | 46.25086 | 8.01800 | 2012 |
| 45 | CH | Valais | 46.57119 | 8.41056 | 2400 |
| 46 | CH | Tessin | 46.56367 | 8.79842 | 1937 |
| 47 | CH | Graubunden | 46.54628 | 9.21139 | 2211 |
| 48 | CH | Graubunden | 46.47153 | 9.72889 | 2262 |
| 49 | CH | Graubunden | 46.58200 | 9.83672 | 2306 |
| 50 | CH | Graubunden | 46.41275 | 10.02408 | 2322 |
| 51 | A | Tyrol | 47.14756 | 10.24817 | 2286 |
| 53 | CH | Graubunden | 46.54856 | 10.43431 | 2456 |
| 54 | I | Lombardia | 46.27239 | 10.57506 | 2303 |
| 55 | A | Tyrol | 46.87197 | 10.70836 | 2557 |
| 57 | A | Tyrol | 47.08960 | 11.54784 | 2313 |
| 58 | I | Trentino Alto Adige/ Südtirol | 46.45667 | 11.88814 | 2117 |
| 59 | I | Trentino Alto Adige/ Südtirol | 46.66434 | 12.18316 | 2391 |
| 66 | I | Trentino Alto Adige/ Südtirol | 46.95562 | 11.51737 | 2101 |
| 69 | I | Trentino Alto Adige/ Südtirol | 46.47751 | 11.81474 | 2297 |
| 73 | A | Tyrol | 47.21906 | 10.31961 | 2180 |
| 74 | A | Osttirol | 46.69904 | 12.44026 | 2117 |
| 75 | CH | Graubunden | 46.52845 | 9.81119 | 2678 |
| 77 | CH | Valais | 46.36692 | 7.65278 | 2259 |
| 78 | CH | Valais | 46.22506 | 7.15989 | 2000 |
| 79 | CH | Graubunden | 46.98753 | 10.35919 | 2280 |
| 80 | CH | Graubunden | 46.66845 | 8.69997 | 2212 |
| 81 | A | Tyrol | 46.98753 | 10.32303 | 2526 |
| 82 | I | Lombardia | 46.53867 | 10.43525 | 2500 |
| 83 | A | Tyrol | 47.04070 | 12.69105 | 2271 |
| 84 | A | Carinthia | 47.06989 | 12.84618 | 2236 |
| 85 | A | Carinthia | 47.05495 | 12.86219 | 2184 |
| 88 | CH | Graubunden | 46.51597 | 9.82125 | 2300 |
| 89 | CH | Graubunden | 46.65067 | 9.75561 | 2265 |
| 90 | CH | Valais | 46.12433 | 7.51753 | 2477 |
| 91 | CH | Valais | 46.56295 | 8.34758 | 2177 |
| 92 | CH | Graubunden | 46.42364 | 9.63836 | 2260 |
| 93 | CH | Valais | 46.20853 | 7.72417 | 2405 |
| 94 | A | Tyrol | 46.99483 | 10.20550 | 2252 |
| 96 | F | Provence-Alpes-Côte d'Azur | 44.81775 | 6.73211 | 2300 |
| 98 | I | Trentino Alto Adige/ Südtirol | 46.48304 | 12.05303 | 2233 |
| 103 | I | Lombardia | 46.49211 | 10.20758 | 2290 |
| 104 | I | Lombardia | 46.45142 | 10.30158 | 2298 |
| 106 | I | Trentino Alto Adige/ Südtirol | 46.94659 | 11.83144 | 2142 |
| 107 | I | Trentino Alto Adige/ Südtirol | 46.51955 | 10.57536 | 2345 |
| 108 | CH | Graubunden | 46.45392 | 9.98694 | 2171 |
| 110 | I | Trentino Alto Adige/ Südtirol | 46.99229 | 12.11355 | 2312 |
| 111 | F | Provence-Alpes-Côte d'Azur | 44.27900 | 6.71922 | 2243 |
| 112 | F | Provence-Alpes-Côte d'Azur | 43.85250 | 6.35278 | 1626 |
| 113 | CH | Graubunden | 46.64370 | 9.86642 | 2446 |
| 114 | F | Provence-Alpes-Côte d'Azur | 44.72089 | 6.91986 | 2339 |
| 115 | F | Provence-Alpes-Côte d'Azur | 44.24500 | 6.75611 | 1891 |
| 116 | F | Provence-Alpes-Côte d'Azur | 44.24797 | 6.76194 | 1953 |
| 117 | F | Provence-Alpes-Côte d'Azur | 43.74556 | 6.65583 | 1632 |
| 118 | I | Piemonte | 44.23161 | 7.62833 | 1636 |
| 119 | I | Piemonte | 44.26778 | 7.21111 | 1820 |
| 120 | I | Piemonte | 44.22333 | 7.12058 | 1966 |
| 121 | F | Provence-Alpes-Côte d'Azur | 44.20639 | 7.11556 | 1710 |
| 200 | I | Piemonte | 44.18431 | 7.60347 | 1390 |
| 201 | F | Provence-Alpes-Côte d'Azur | 44.08044 | 6.63750 | 2025 |
| 202 | F | Provence-Alpes-Côte d'Azur | 44.15900 | 6.71461 | 1829 |
| 203 | F | Provence-Alpes-Côte d'Azur | 44.16294 | 6.71081 | 1840 |
| 204 | F | Provence-Alpes-Côte d'Azur | 44.15725 | 6.72920 | 1640 |
| 205 | F | Provence-Alpes-Côte d'Azur | 44.23711 | 6.70478 | 2265 |
| 206 | F | Provence-Alpes-Côte d'Azur | 44.24680 | 6.69880 | 2049 |
| 207 | F | Provence-Alpes-Côte d'Azur | 44.30108 | 6.56739 | 1921 |
| 208 | F | Provence-Alpes-Côte d'Azur | 44.30244 | 6.56097 | 1924 |
| 209 | F | Provence-Alpes-Côte d'Azur | 44.28967 | 6.74344 | 1942 |
| 210 | F | Provence-Alpes-Côte d'Azur | 44.36180 | 6.78758 | 2060 |
| 211 | F | Provence-Alpes-Côte d'Azur | 44.32505 | 6.85761 | 1983 |
| 212 | F | Provence-Alpes-Côte d'Azur | 44.33314 | 6.87167 | 2258 |
| 213 | F | Provence-Alpes-Côte d'Azur | 44.42606 | 6.88950 | 1951 |
| 214 | F | Provence-Alpes-Côte d'Azur | 44.41444 | 6.88917 | 2020 |
| 230 | I | Piemonte | 44.15920 | 7.57209 | 1581 |
| 231 | F | Provence-Alpes-Côte d'Azur | 44.09426 | 7.35626 | 1873 |
| 232 | F | Provence-Alpes-Côte d'Azur | 44.09839 | 7.31020 | 2122 |
| 233 | F | Provence-Alpes-Côte d'Azur | 44.12946 | 6.96878 | 2185 |
| 234 | F | Provence-Alpes-Côte d'Azur | 44.16116 | 6.71292 | 1805 |
| 235 | F | Provence-Alpes-Côte d'Azur | 44.16689 | 6.70763 | 1930 |
